# Prevalence and assemblage distribution of *Giardia intestinalis* in farmed mink, foxes, and raccoon dogs in northern China

**DOI:** 10.3389/fvets.2025.1514525

**Published:** 2025-02-19

**Authors:** Shuo Liu, Miao Zhang, Nian-Yu Xue, Hai-Tao Wang, Zhong-Yuan Li, Ya Qin, Xue-Min Li, Qing-Yu Hou, Jing Jiang, Xing Yang, Hong-Bo Ni, Jian-Xin Wen

**Affiliations:** ^1^College of Veterinary Medicine, Qingdao Agricultural University, Qingdao, China; ^2^College of Life Sciences, Changchun Sci-Tech University, Shuangyang, China; ^3^College of Veterinary Medicine, Yangzhou University, Yangzhou, China; ^4^Guangxi Key Laboratory of Brain and Cognitive Neuroscience, College of Basic Medicine, Guilin Medical University, The Guangxi Zhuang Autonomous Region, Guilin, China; ^5^College of Animal Science and Technology, Jilin Agricultural University, Changchun, China; ^6^Department of Medical Microbiology and Immunology, School of Basic Medicine, Dali University, Dali, China

**Keywords:** prevalence, *Giardia intestinalis*, assemblage, mink, raccoon dog

## Abstract

*Giardia intestinalis* is a widespread protozoan parasite associated with significant health risks in humans and animals. However, there is a lack of epidemiological data regarding this parasite in fur-animals. The present study aimed to investigate the prevalence and assemblage distribution of *G. intestinalis* in fur-animals in northern China. A total of 871 fecal samples were detected by nested PCR. The results showed an overall infection rate of 1.15%, with the highest rate in Hebei province (2.28%), while no positive cases were observed in Jilin and Heilongjiang provinces. Although no significant differences were found in species group, raccoon dogs (1.72%) were more susceptible to infection than mink (1.40%) and foxes (0.57%). Additionally, the highest infection rate was observed in farms with fewer than 2,000 animals (1.41%), followed by farms with ≥5,000 (0.93%) and those with 2,000–5,000 animals (0.75%). The infection rate was higher in juvenile animals (1.35%) compared to adults (1.08%), and in non-diarrheal animals (1.16%) compared to diarrheal animals (1.08%). Notably, this study is the first to report assemblage A in mink, this finding highlight the potential role of mink as a reservoir for zoonotic transmission. Assemblage D was detected in foxes and raccoon dogs, further suggesting that these animals may serve as potential zoonotic reservoirs. These findings not only complements the epidemiological data of *G. intestinalis* in fur-animals but also emphasize the importance of monitoring the fur industry to mitigate public health risks.

## 1 Introduction

*Giardia intestinalis* (Syn. *G. duodenalis* or *G. lamblia*) is a flagellated protozoan parasite that widely infects the intestines of humans and various animals (1). It spreads through direct and indirect contact (via food and water) (2). Globally, *G. intestinalis* is one of the leading pathogens responsible for parasitic-related diarrhea, with infection rates strongly correlated with regional sanitation conditions. The prevalence in developed countries is significantly lower than in developing countries (3). The World Health Organization (WHO) estimated that approximately 280 million people are infected with this parasite annually, resulting in acute diarrhea (4). Additionally, individuals infected with *G. intestinalis* may face long-term health risks, including irritable bowel syndrome, childhood malnutrition, and arthritis (5–7). Furthermore, the rise of cross-border animal trade complicates public health control efforts, blurring the lines of parasite transmission and increasing the urgency for effective global surveillance and management strategies.

Molecular biology techniques have been widely applied in the study of *G. intestinalis*. Common methods include small subunit ribosomal RNA (*SSU* rRNA) analysis and multilocus genotyping (*gdh*, *tpi*, and *bg* genes) (8). Through molecular analysis, *G. intestinalis* has been divided into eight assemblages (A–H) (9). Among these, assemblages A and B exhibit strong cross-host transmission capabilities, infecting diverse hosts, including humans, and are typical zoonotic pathogens (10–12). In contrast, assemblages C–H show distinct host specificity. Studies have shown that assemblages C and D primarily infect canines (10, 13), assemblage E mainly infects ungulates such as cattle and sheep (14, 15), assemblages F and H have been detected in marine animals (16, 17), assemblage G was specifically found in rodents (15, 18).

Interestingly, despite assemblages C and D typically being considered exclusive to canines, research has shown some exceptions. Assemblage D was detected in a German traveller, and a study in Egypt identified assemblage C as a zoonotic pathogen (19, 20). These findings suggest that the host range of *G. intestinalis* may be broader than previously known. Therefore, expanding our understanding of its host adaptability is crucial for effective disease control and prevention strategies.

The fur animal farming industry in northern China is large-scale and is one of the pillars of the local economy. However, with the development of large-scale farming, disease prevention and control face significant challenges. Research has shown that fur animals can be infected with various parasites, including *Pentatrichomonas hominis*, *Sarcocystis* spp., and *Trichinella spiralis* (21–23), indicating that fur animals may be potential hosts for zoonotic pathogens. However, reports on the infection of fur animals (mink, foxes, and raccoon dogs) with *G. intestinalis* are scarce globally. This study employs *tpi*, *gdh*, and *bg* gene analysis to test samples from mink, foxes, and raccoon dogs, aiming to investigate the infection rates of *G. intestinalis* and the diversity and distribution of assemblage in these animals as well as to assess the implications for public health and disease prevention in the farming industry.

## 2 Materials and methods

### 2.1 Sample collection

**Figure 1:**
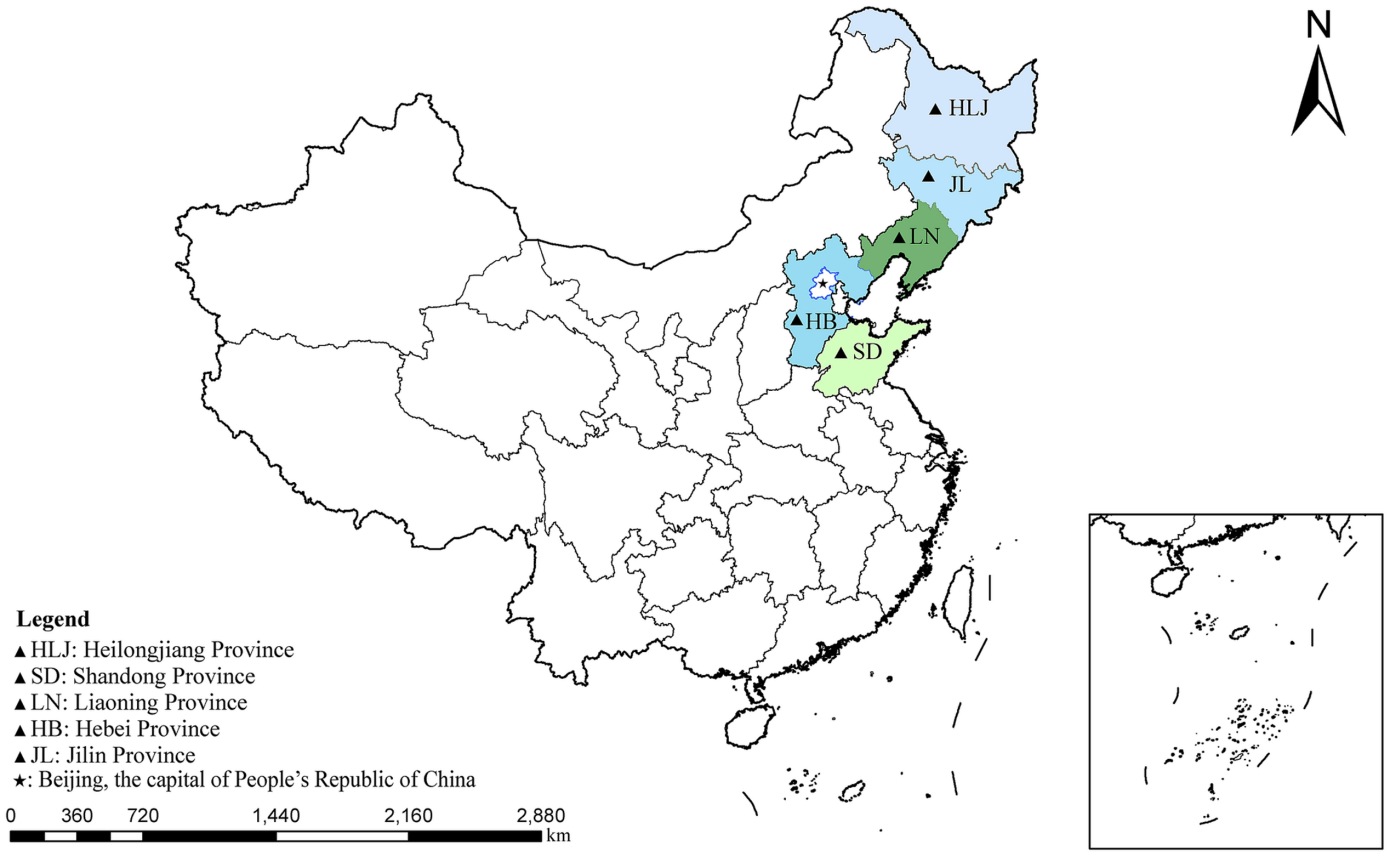
A map of the People’s Republic of China showing the sampling regions marked as triangles.

From October 2023 to May 2024, 871 fresh fecal samples were randomly collected from farmed fur animals in main fur farming provinces of China (Shandong, Hebei, Jilin, Liaoning, and Heilongjiang). The samples included mink (*n* = 286), foxes (*n* = 352), and raccoon dogs (*n* = 233) (Figure 1). All animals are self-breed and kept in cages, foxes and raccoon dogs are breed in individual cages, while minks are breed in group cages with three to five animals. No direct contact with animals, fresh feces were collected immediately from beneath the cages using disposable PE gloves when the animal excreted feces. Detailed records of sample’s source animal, sample ID, sampling position, date, animal age, health status, species, and farm size. To maintain sample integrity, samples were stored in 12 mL collection tubes, transported to the laboratory on dry ice, and preserved at −80°C.

### 2.2 DNA extraction and PCR amplification

According to the manufacturer’s instructions, genomic DNA was extracted from each sample using the E.Z.N.A.^®^ Stool DNA Kit (Omega Biotek, Norcross, GA, United States) and stored at −20°C until PCR analysis. First, the β-giardin (*bg*) gene was amplified using nested PCR to confirm the presence of *G. intestinalis* in the samples. Subsequently, the positive samples were subjected to amplification of the glutamate dehydrogenase (*gdh*) and triosephosphate isomerase (*tpi*) genes (24–26). The first round was performed with a 25 μL reaction mixture containing 12.5 μL of premix enzyme (dNTPs, DNA polymerase, buffer and Mg^2+^), 8.5 μL of ddH_2_O, 1 μL of forward primer, 1 μL of reverse primer, and 2 μL of template DNA. In the second round, 2 μL of the first-round product was mixed with 25 μL of premix enzyme, 21 μL of ddH_2_O, 1 μL of forward primer, and 1 μL of reverse primer, in a total volume of 50 μL. Then, 5 μL of the product was tested using 1% agarose gel electrophoresis. Positive PCR products were sequenced by Anhui General Biosystems Co., Ltd. (Anhui China) using Sanger sequencing.

### 2.3 Sequence and phylogenetic analysis

The sequences of the *bg* and *gdh* genes were obtained from Anhui General Corporation. BLAST^1^ was used to align these sequences with the corresponding *bg*, and *gdh* reference sequences in GenBank. Phylogenetic trees were constructed using the neighbour-joining (NJ) method in MEGA11 software^2^ to study the relationships between different isolates and to illustrate the genetic diversity of *G. intestinalis* (27). The reliability of the phylogenetic analysis was evaluated through 1,000 bootstrap replicates.

### 2.4 Statistical analysis

Chi-square analysis in SAS (v9.0) software was used to evaluate the impact of sampling region (*x*1), species (*x*2), farming scale (*x*3), health status (*x*4), and age (*x*5) on the infection rate of *G. intestinalis* (*y*). In the multivariable regression analysis, each variable was individually included in the binary logistic model. The best model was selected using the Fisher scoring method. In the Statistical Product and Service Solutions (SPSS, IBM Corp., Armonk, NY, United States) software, chi-square tests were performed to explore the differences in the prevalence of *G. intestinalis* across various study factors while calculating the odds ratio (OR) and 95% confidence interval (95% CI). A *p*-value of less than 0.05 was considered statistically significant.

## 3 Results

### 3.1 Prevalence of *Giardia intestinalis* in mink, raccoon dogs, and foxes across the five provinces

**Figure 2:**
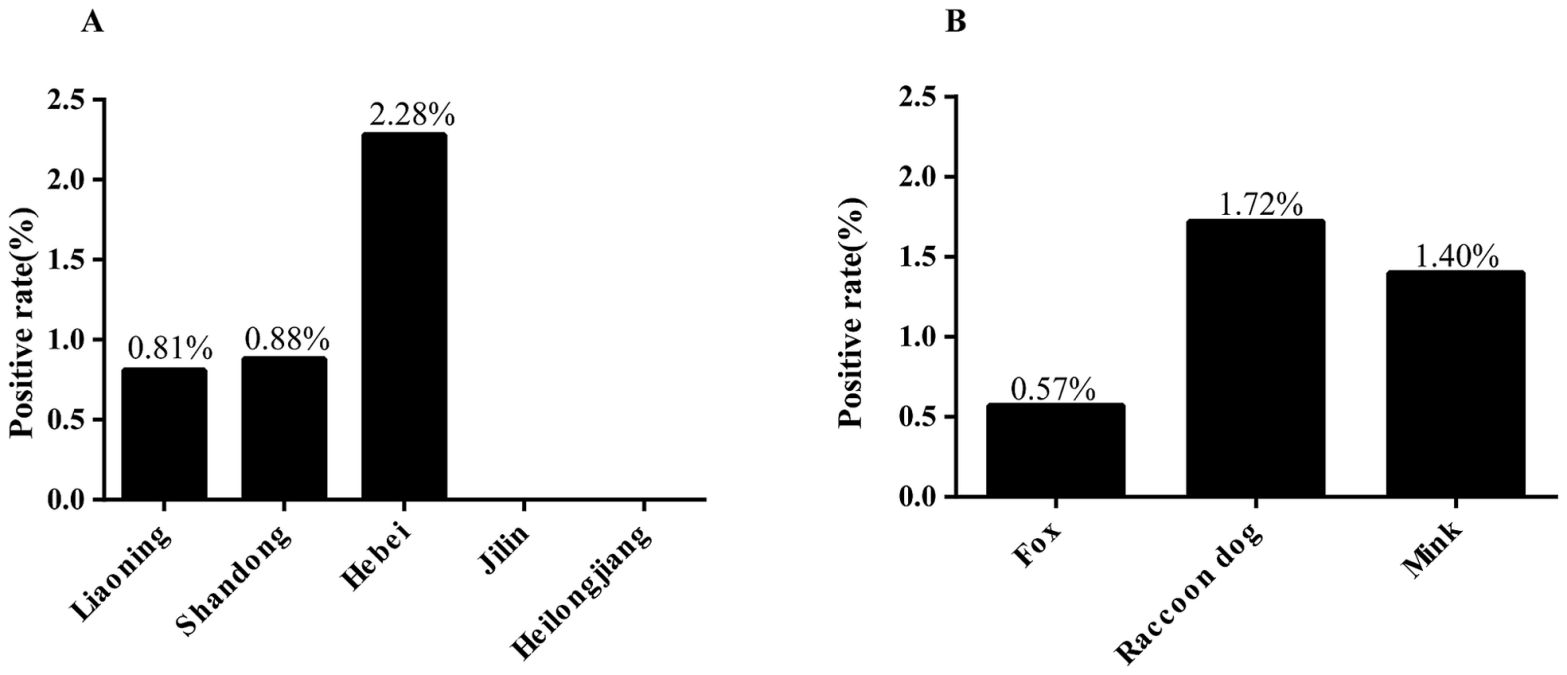
Infection rate of *G. intestinalis* in fur-animals under various factors. **(A)** Infection rate of *G. intestinalis* in fur-animals in different provinces. **(B)** Infection rate of *G. intestinalis* in different species.

In the present study, 10 (1.15%) positive cases of *G. intestinalis* were detected through nested PCR from 871 fecal samples. The differences in infection rates among provinces were not statistically significant (*χ*^2^ = 5.88, df = 4, *p* = 0.2082). Hebei province had the highest infection rate (2.28%, 7/307, OR = 2.86 95% CI 0.59–13.88), followed by Shandong province (0.88%, 1/113, OR = 1.09 95% CI 0.10–12.19) and Liaoning province (0.81%, 2/247), while no positive cases were observed in Jilin and Heilongjiang provinces (Table 1 and Figure 2A). Similarly, the differences in infection rates between species were not statistically significant (*χ*^2^ = 2, df = 2, *p* = 0.3680). Raccoon dogs had the highest infection rate (1.72%, 4/233, OR = 3.06 95% CI 0.56–16.83), followed by mink (1.40%, 4/286, OR = 2.48 95% CI 0.45–13.65) and foxes (0.57%, 2/352) (Table 1 and Figure 2B).

**Table 1:** Prevalence of *G. intestinalis* determined by sequence analysis of the *bg* gene.

Variables	Categories	No. positive /No. test	% (95% CI)	Heterogeneity	OR (95% CI)
				*χ*^2^/df/*I*^2^(%)/*p*	
Region	Liaoning province	2/247	0.81 (0.01–2.42)	5.88/4/32/0.2082	Reference
	Shandong province	1/113	0.88 (0.00–3.76)		1.09 (0.10–12.19)
	Hebei province	7/307	2.28 (0.86–4.30)		2.86 (0.59–13.88)
	Jilin province	0/73	0.00 (—)		—
	Heilongjiang province	0/131	0.00 (—)		—
Species	Fox	2/352	0.57 (0.01–1.70)	2.00/2/0/0.3680	Reference
	Raccoon dog	4/233	1.72 (0.37–3.87)		3.06 (0.56–16.83)
	Mink	4/286	1.40 (0.30–3.16)		2.48 (0.45–13.65)
Farm size	2,000–5,000	2/266	0.75 (0.01–2.25)	0.49/2/0/0.7837	Reference
	<2,000	7/498	1.41 (0.53–2.66)		1.88 (0.39–9.12)
	≥5,000	1/107	0.93 (0.00–3.97)		1.25 (0.11–13.88)
Age	Adult	7/649	1.08 (0.40–2.04)	0.21/1/0/0.6481	Reference
	Juvenile	3/222	1.35 (0.17–3.40)		1.26 (0.32–4.90)
Condition	Diarrheal	1/93	1.08 (0.00–4.56)	0.07/1/0/0.7947	Reference
	Non-diarrheal	9/778	1.16 (0.51–2.05)		1.08 (0.14–8.60)
Total	—	10/871	1.15 (0.55–2.10)	—	—

There was no statistically significant difference in the farm size group (*χ*^2^ = 0.49, df = 2, *p* = 0.7837), with the lowest infection rate was observed in farms with 2,000 to 5,000 animals (0.75%, 2/266), the highest infection rate was found in farms with fewer than 2,000 animals (1.41%, 7/498, OR = 1.88 95% CI 0.39–9.12), and the infection rate of farms with more than 5,000 was 0.94% (1/107, OR = 1.25 95% CI 0.11–13.88). Additionally, the infection rate in adult animals (1.08%, 7/649) was slightly lower than in juvenile animals (1.35%, 3/222, OR = 1.26 95% CI 0.32–4.90), the difference was not statistically significant (*χ*^2^ = 0.21, df = 1, *p* = 0.6481). The infection rate in non-diarrheal animals (1.16%, 9/778 OR = 1.08 95% CI 0.14–8.60) was slightly higher than in diarrheal animals (1.08%, 1/93), and the statistical difference was not significant (*χ*^2^ = 0.07, df = 1, *p* = 0.7947) (Table 1).

### 3.2 Influencing factors

The present study evaluated the influencing factors affecting the infection rate of *G. intestinalis* using logistic forward stepwise regression analysis. The final best model included sampling region and health status. The model equation is *y* = −7.2133*x*1 + 0.8197*x*2 + 3.8666.

The results show that the sampling area negatively affects the infection rate of *G. intestinalis* (OR = 0.74, 95% CI 0.09–1.81). Hebei had the highest infection rate (OR = 2.86, 95% CI 0.59–13.88), followed by Shandong (OR = 1.09, 95% CI 0.10–12.19) and Liaoning (0.81%, 2/247). No infections were observed in Jilin (OR = 0.67, 95% CI 0.03–14.07) and Heilongjiang (OR = 0.37, 95% CI 0.02–7.84). Health status positively influences infection (OR = 0.98, 95% CI 0.36–1.82), and the infection rate in diarrheal animals (1.08%, 1/93) was lower than in non-diarrheal animals (OR = 1.08, 95% CI 0.14–8.60) (Table 1).

### 3.3 Assemblage of *Giardia intestinalis* determined through *bg* and *gdh* sequence analysis

The present study conducted nested PCR detection on 871 fecal samples, identifying 10 positive samples for the *bg* gene and obtaining six assembled sequences. Among these, three samples from mink belonged to assemblage A. Additionally, one sample from foxes and two from raccoon dogs were classified as assemblage D (Table 2).

**Table 2:** Prevalence and assemblage distribution of *G. intestinalis*.

Variables	Categories	No. positive/No. test (%)	Assemblage of *G. intestinalis* (*n*)	
			*bg*	*gdh*
Region	Liaoning province	2/247 (0.81%)	0	0
	Shandong province	1/113 (0.88%)	0	D (1)
	Hebei province	7/307 (2.28%)	A (3); D (3)	A (3); D (2)
	Jilin province	0/73 (0%)	0	0
	Heilongjiang province	0/131 (0%)	0	0
Species	Fox	2/352 (0.57%)	D (1)	D (2)
	Raccoon dog	4/233 (1.72%)	D (2)	D (1)
	Mink	4/286 (1.40%)	A (3)	A (3)
Raising scale	2,000–5,000	2/266 (0.75%)	D (1)	D (1)
	<2,000	7/498 (1.41%)	A (3); D (2)	A (3); D (2)
	≥5,000	1/107 (0.93%)	0	0
Age	Adult	7/649 (1.08%)	A (3); D (2)	A (3); D (2)
	Juvenile	3/222 (1.35%)	D (1)	D (1)
Condition	Diarrheal	1/93 (1.08%)	0	0
	Non-diarrheal	9/778 (1.16%)	A (3); D (3)	A (3); D (3)
Total	—	10/871 (1.15%)	A (3); D (3)	A (3); D (3)

We further tested the *bg* gene-positive samples to amplify the *gdh* gene, successfully obtaining six *gdh* sequences. Analysis of the *gdh* gene sequences showed that three mink samples belonged to assemblage A as well as two fox samples and one raccoon dog sample belonged to assemblage D. Meanwhile, we detected the *tpi* gene, but failed to obtain any sequences (Table 2).

**Figure 3:**
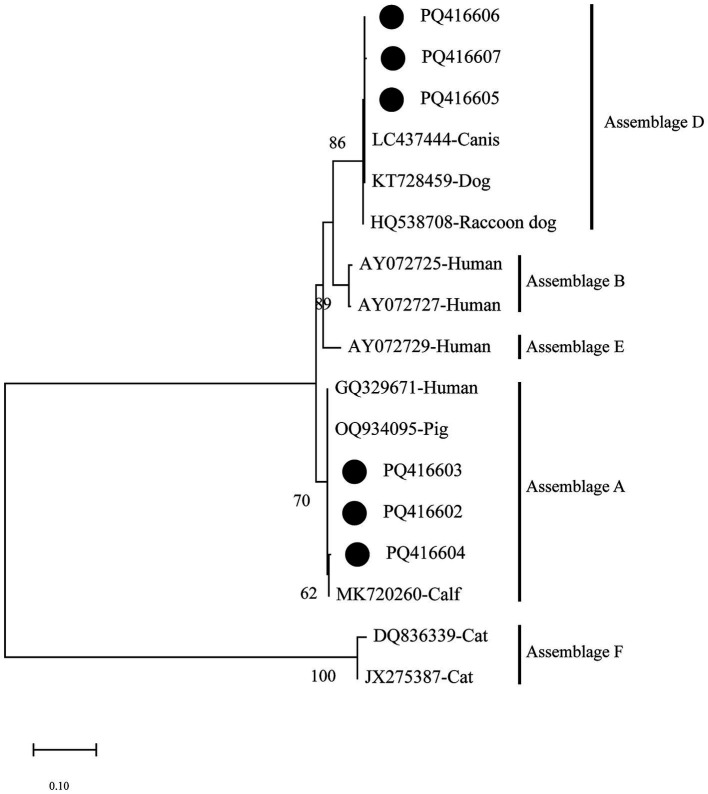
Evolutionary relationships among *Giardia intestinalis* inferred by neighbour-joining analysis using the Kimura 2-parameter model of *bg* gene sequences. Numbers on branches are percent bootstrap values from 1,000 replicates, with only those greater than 50% shown. The sequences obtained in the present study are represented by black dots.

Based on the phylogenetic analysis of the *bg* gene, the results indicated that sequences PQ416602–PQ416604 primarily cluster in the branch of assemblage A. PQ416604 is grouped with MK720260 (Calf) in the same branch, forming a sister group with PQ416602, PQ416603, and GQ329671 (Human). Meanwhile, sequences PQ416605–PQ416607 cluster with LC437444 (Canis) in the branch of assemblage D, demonstrating a high degree of phylogenetic relationship (Figure 3).

**Figure 4:**
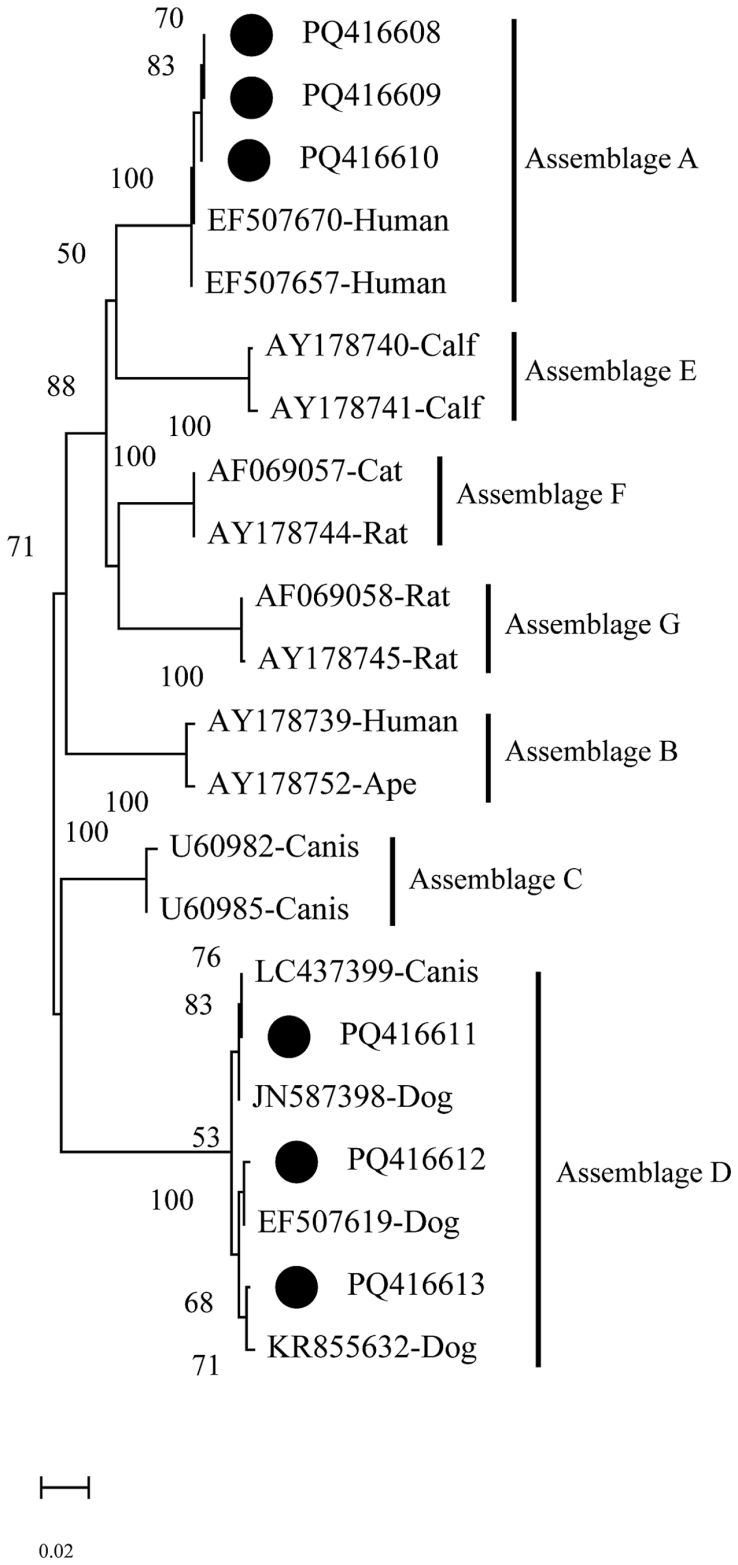
Evolutionary relationships among *Giardia intestinalis* inferred by neighbour-joining analysis using the Kimura 2-parameter model of *gdh* gene sequences. Numbers on branches are percent bootstrap values from 1,000 replicates, with only those greater than 50% shown. The sequences obtained in the present study are represented by black dots.

The phylogenetic analysis of the *gdh* gene shows that PQ416608–PQ416610 cluster in the branch of assemblage A and group together with EF507670 (Human) and EF507657 (Human), exhibiting a 100% bootstrap support value, indicating a high degree of phylogenetic relationship with human-derived *Giardia*. On the other hand, sequences PQ416611–PQ416613 mainly cluster in the branch of assemblage D, where they group with LC437399 (Canis), EF507619 (Dog), and KR855632 (Dog), indicating a close genetic similarity with the *Giardia* assemblages of canine hosts (Figure 4).

## 4 Discussion

Foodborne zoonotic diseases are a significant global public health issue, especially in regions with frequent agricultural and livestock activities. These diseases not only threaten animal health but also pose potential risks to human health, particularly when the sources of transmission are complex (28). *G. intestinalis*, a typical foodborne zoonotic pathogen, has been confirmed to transmit between humans and various animals, and it poses a severe threat to immunocompromised individuals (29). Therefore, studying the epidemiological characteristics of this parasite is of utmost importance. In this study, we tested farmed fur-animals in northern China, and the overall prevalence of *G. intestinalis* in fur-animals was 1.15% (10/871), which was lower than other animal populations in China, such as cattle in Shanxi province (28.3%, 243/858), dogs in Liaoning province (13.2%, 27/205), black bears in Heilongjiang province (8.3%, 3/36), and donkeys in Jilin, Lioning, and Shandong provinces (15.5%, 28/181) (13, 30–33). The difference may be related to species differences, sample size and living habits. Interestingly, in this study, animals showed different susceptibilities to the disease, raccoon dogs had the highest infection rate (1.72%, 4/233), followed by mink (1.40%, 4/286) and foxes (0.57%, 2/352). although, no significant differences were found among them, which may be related to the small sample size. Future studies could increase the sample size and further investigate the infection mechanisms of *G. intestinalis* to difference species.

In the present study, the sampling region negatively affected the infection rates of *G. intestinalis*. The highest infection rate was observed in Hebei province (2.28%, 7/307), followed by Shandong province (0.88%, 1/113) and Liaoning province (0.81%, 2/247). Notably, no positive cases were detected in Jilin (0/73) and Heilongjiang (0/131) provinces, suggesting a lower risk of infection in these regions. However, a report showed that the infection rate of *G. intestinalis* in diarrhea patients in Heilongjiang was 5.81%, which indicated that the epidemic prevention and control situation was still not optimistic (31). Future studies should aim to expand both the sample size and the range of animal testing. Furthermore, the present study also found that the health status of animals was an important influencing factor affecting the infection rate of *G. intestinalis*. The infection rate in non-diarrheal animals was 1.16% (9/778) higher than the 1.08% (1/93) observed in diarrheal animals. This contrasts with findings in cattle in Shanxi province and children in Zhejiang province (30, 34). This difference may be related to the stages of *G. intestinalis* infection and the pathogenic mechanisms that cause diarrhea. Additionally, the host’s age, immune status, and co-infections with other pathogens may also influence the clinical manifestations and transmission patterns of *G. intestinalis* infection.

In this study, no significant differences in infection rates were observed between farms with different farming sizes. Notably, the infection rate of farms with less than 2,000 animals (1.41%, 7/498) was higher than that of farms with 2,000–5,000 animals (0.75%, 2/266) and ≥5,000 animals (0.93%, 1/107). These small-scale farms are usually family-run, and the technical level of feeding management, disease prevention and control is relatively low. Additionally, some farms have the phenomenon of raising dogs together, which may be a factor leading to the high infection rate.

Researchers have classified *G. intestinalis* into eight genotype assemblages (A–H), based on common genetic markers such as the *SSU* rRNA gene, and the *gdh*, *bg*, and *tpi* loci (9). In the present study, *G. intestinalis* in fur-animals were identified primarily as assemblages A (*n* = 3) and D (*n* = 3) through the analysis of *bg* and *gdh* genes. Among them, assemblage A was the dominant assemblage in mink, consistent with studies on ferrets in Europe and Japan, confirming that assemblage A was common in mustelids (35–37). Assemblage A is distributed globally across mammals and poultry and is recognised as an important pathogen responsible for human infections (3, 9, 33, 38). The discovery of assemblage A in mink in this study indicates that mink may be a potential host for human infections. Notably, pathological reports indicate that both person-to-person and person-to-animal transmission can occur through direct contact (49). Therefore, it is recommended to further investigate the infection rates among farmers and industry workers to prevent the spread to other animal populations or human communities.

Assemblages C and D were the most common genotypes in canids globally (9). They were widely reported in dogs (13, 39, 40). In China, these assemblages have also been detected in dogs from Xinjiang and raccoon dogs from Jilin (41). In the present study, assemblage D was found in foxes and raccoon dogs, consistent with studies on foxes in Australia and raccoon dogs in Poland (42, 43). However, assemblage C was not detected in this study, which may be related to sample size, sampling time, and geographic regional differences. Notably, although assemblage D was considered host-specific to canids, existing research shows that it can infect other species, including humans (9). For instance, assemblage D has been detected in German travellers, Australian kangaroos, British cattle, and American chinchillas (19, 37, 43, 44). These findings suggest that assemblage D may cross-infection between wildlife and livestock, posing a potential zoonotic risk.

In this study, the amplification success rates for the *bg* and *gdh* genes were relatively high, whereas the *tpi* gene sequences were not successfully obtained. This suggests that the amplification success rates for *bg*, *gdh*, and *tpi* genes may be associated with different assemblage types, which is consistent with previous studies. For example, Chen et al. (31) successfully obtained 20 *bg*, 19 *gdh*, and 9 *tpi* sequences from 22 positive samples of black bears, while Xiao et al. (45) obtained 70 *bg*, 32 *gdh*, and 7 *tpi* sequences from 90 positive samples of goats. These results indicate that more accurate and sensitive molecular diagnostic techniques are urgently needed to achieve a more precise genetic characterization of *G. intestinalis.*

*G. intestinalis* exists as cysts in vegetables, meat and other foods (29). The food source of fur-animals in mainly poultry meat, which may be one of their infection routes. Additionally, *G. intestinalis* is also wildly present in the environment, particularly in surface water sources (46). In 2013, a waterborne outbreak of *G. intestinalis* infection was reported in South Korea, highlighting its potential for waterborne transmission (47). In addition to known hosts, there are many unknown hosts that could serve as potential sources of human infection. These potential transmission routes pose a threat to public health security. Therefore, it is essential to strengthen the quarantine of foods such as vegetables and meat, while expanding the sampling scope to include more species for detection. At the same time, treatment of the disease is also crucial. Although metronidazole is the drug of choice for treating giardiasis, its issues with resistance and potential side effects such as abdominal pain and nausea limit its use. Therefore, there is an urgent need to develop new treatment formulations, such as phytochemicals, to address this challenge (48).

## 5 Conclusion

The present study investigated the prevalence of *G. intestinalis* in fur-animals in northern China, first reporting the occurrence of assemblage A in mink, indicating that mink may be a potential zoonotic source of *G. intestinalis*, while also contributing to the epidemiological data on this parasite. Additionally, the detection of assemblage D in foxes and raccoon dogs suggests a similar zoonotic risk. Therefore, attention should be given to the prevalence of *G. intestinalis* among fur industry workers and their surrounding environments to effectively control and prevent potential transmission risks. However, this study has some limitations. For instance, the *tpi* gene sequence was not successfully obtained, and the effect of seasonality on prevalence was not investigated. Therefore, future studies should aim to expand the sample size and incorporate seasonal sampling to better understand the infection dynamics of *G. intestinalis* in fur-animals.

## Data Availability

The datasets presented in this study can be found in online repositories. The names of the repository/repositories and accession number(s) can be found below: https://www.ncbi.nlm.nih.gov/genbank/, PQ416602-PQ416613.

## Footnotes

^1^ https://blast.ncbi.nlm.nih.gov/

^2^ http://www.megasoftware.net/

## References

[1] Huang DB White AC. An updated review on *Cryptosporidium* and *Giardia*. Gastroenterol Clin N Am. (2006) 35:291–314. doi: 10.1016/j.gtc.2006.03.006 16880067

[2] Drake J Sweet S Baxendale K Hegarty E Horr S Friis H. Detection of *Giardia* and helminths in Western Europe at local K9 (canine) sites (DOGWALKS Study). Parasit Vectors. (2022) 15:311. doi: 10.1186/s13071-022-05440-2 36057606 PMC9440314

[3] Feng Y Xiao L. Zoonotic potential and molecular epidemiology of *Giardia* species and giardiasis. Clin Microbiol Rev. (2011) 24:110–40. doi: 10.1128/CMR.00033-10 21233509 PMC3021202

[4] Einarsson E Ma’ayeh S Svärd SG. An update on *Giardia* and giardiasis. Curr Opin Microbiol. (2016) 34:47–52. doi: 10.1016/j.mib.2016.07.019, PMID: 27501461

[5] Cook DM Swanson RC Eggett DL Booth GM. A retrospective analysis of the prevalence of gastrointestinal parasites among school children in the Palajunoj Valley of Guatemala. J Health Popul Nutr. (2009) 27:31–40. doi: 10.3329/jhpn.v27i1.3321 19248646 PMC2761809

[6] Krol A. *Giardia lamblia* as a rare cause of reactive arthritis. Ugeskr Laeger. (2013) 175:V05130347 PMID: 25353256

[7] Allain T Buret AG. Pathogenesis and post-infectious complications in giardiasis. Adv Parasitol. (2020) 107:173–99. doi: 10.1016/bs.apar.2019.12.001 32122529

[8] Qi M Dong H Wang R Li J Zhao J Zhang L. Infection rate and genetic diversity of *Giardia duodenalis* in pet and stray dogs in Henan province, China. Parasitol Int. (2016) 65:159–62. doi: 10.1016/j.parint.2015.11.008, PMID: 26631754

[9] Heyworth MF. *Giardia duodenalis* genetic assemblages and hosts. Parasite. (2016) 23:13. doi: 10.1051/parasite/2016013, PMID: 26984116 PMC4794627

[10] Berrilli F D’Alfonso R Giangaspero A Marangi M Brandonisio O Kaboré Y. *Giardia duodenalis* genotypes and *Cryptosporidium* species in humans and domestic animals in Côte d’Ivoire: occurrence and evidence for environmental contamination. Trans R Soc Trop Med Hyg. (2012) 106:191–5. doi: 10.1016/j.trstmh.2011.12.005 22265078

[11] Covacin C Aucoin DP Elliot A Thompson RC. Genotypic characterisation of *Giardia* from domestic dogs in the USA. Vet Parasitol. (2011) 177:28–32. doi: 10.1016/j.vetpar.2010.11.029 21146935

[12] Traversa D Otranto D Milillo P Latrofa MS Giangaspero A Cesare AD. *Giardia duodenalis* sub-assemblage of animal and human origin in horses. Infect Genet Evol. (2012) 12:1642–6. doi: 10.1016/j.meegid.2012.06.014 22771626

[13] Li W Liu C Yu Y Li J Gong P Song M. Molecular characterization of *Giardia duodenalis* isolates from police and farm dogs in China. Exp Parasitol. (2013) 135:223–6. doi: 10.1016/j.exppara.2013.07.009 23891941

[14] Khan SM Debnath C Pramanik AK Xiao L Nozaki T Ganguly S. Molecular evidence for zoonotic transmission of *Giardia duodenalis* among dairy farm workers in West Bengal, India. Vet Parasitol. (2011) 178:342–5. doi: 10.1016/j.vetpar.2011.01.029 21324592

[15] Lebbad M Mattsson JG Christensson B Ljungström B Backhans A Andersson JO. From mouse to moose: multilocus genotyping of *Giardia* isolates from various animal species. Vet Parasitol. (2010) 168:231–9. doi: 10.1016/j.vetpar.2009.11.003 19969422

[16] Lasek-Nesselquist E Welch DM Sogin ML. The identification of a new *Giardia duodenalis* assemblage in marine vertebrates and a preliminary analysis of *G. Duodenalis* population biology in marine systems. Int J Parasitol. (2010) 40:1063–74. doi: 10.1016/j.ijpara.2010.02.015 20361967 PMC2900473

[17] Reboredo-Fernández A Ares-Mazás E Martínez-Cedeira JA Romero-Suances R Cacciò SM Gómez-Couso H. *Giardia* and *Cryptosporidium* in cetaceans on the European Atlantic coast. Parasitol Res. (2015) 114:693–8. doi: 10.1007/s00436-014-4235-8, PMID: 25418072

[18] Zhao Z Wang R Zhao W Qi M Zhao J Zhang L. Genotyping and subtyping of *Giardia* and *Cryptosporidium* isolates from commensal rodents in China. Parasitology. (2015) 142:800–6. doi: 10.1017/S0031182014001929 25579244

[19] Broglia A Weitzel T Harms G Cacció SM Nöckler K. Molecular typing of *Giardia duodenalis* isolates from German travellers. Parasitol Res. (2013) 112:3449–56. doi: 10.1007/s00436-013-3524-y, PMID: 23892479

[20] Soliman RH Fuentes I Rubio JM. Identification of a novel assemblage B subgenotype and a zoonotic assemblage C in human isolates of *Giardia intestinalis* in Egypt. Parasitol Int. (2011) 60:507–11. doi: 10.1016/j.parint.2011.09.006 21989040

[21] Song P Guo Y Zuo S Li L Liu F Zhang T. Prevalence of *Pentatrichomonas hominis* in foxes and raccoon dogs and changes in the gut microbiota of infected female foxes in the Hebei and Henan provinces in China. Parasitol Res. (2023) 123:74. doi: 10.1007/s00436-023-08099-5, PMID: 38155301

[22] Máca O Gudiškis N Butkauskas D González-Solís D Prakas P. Red foxes (*Vulpes vulpes*) and raccoon dogs (*Nyctereutes procyonoides*) as potential spreaders of *Sarcocystis* species. Front Vet Sci. (2024) 11:1392618. doi: 10.3389/fvets.2024.1392618 38903682 PMC11188440

[23] Zhang NZ Li WH Yu HJ Liu YJ Qin HT Jia WZ. Novel study on the prevalence of *Trichinella spiralis* in farmed American minks (*Neovison vison*) associated with exposure to wild rats (*Rattus norvegicus*) in China. Zoonoses Public Health. (2022) 69:938–43. doi: 10.1111/zph.12991 36345967

[24] Lalle M Pozio E Capelli G Bruschi F Crotti D Cacciò SM. Genetic heterogeneity at the beta-giardin locus among human and animal isolates of *Giardia duodenalis* and identification of potentially zoonotic subgenotypes. Int J Parasitol. (2005) 35:207–13. doi: 10.1016/j.ijpara.2004.10.022 15710441

[25] Cacciò SM Beck R Lalle M Marinculic A Pozio E. Multilocus genotyping of *Giardia duodenalis* reveals striking differences between assemblages a and B. Int J Parasitol. (2008) 38:1523–31. doi: 10.1016/j.ijpara.2008.04.008, PMID: 18571176

[26] Sulaiman IM Fayer R Bern C Gilman RH Trout JM Schantz PM. Triosephosphate isomerase gene characterization and potential zoonotic transmission of *Giardia duodenalis*. Emerg Infect Dis. (2003) 9:1444–52. doi: 10.3201/eid0911.030084 14718089 PMC3035538

[27] Tamura K Stecher G. MEGA11: molecular evolutionary genetics analysis version 11. Mol Biol Evol. (2021) 38:3022–7. doi: 10.1093/molbev/msab120 33892491 PMC8233496

[28] Murrell KD. Zoonotic foodborne parasites and their surveillance. Rev Sci Tech. (2013) 32:559–69. doi: 10.20506/rst.32.2.2239 24547659

[29] Ryan U Hijjawi N Feng Y Xiao L. Giardia: an under-reported foodborne parasite. Int J Parasitol. (2019) 49:1–11. doi: 10.1016/j.ijpara.2018.07.003 30391227

[30] Zhao L Wang Y Wang M Zhang S Wang L Zhang Z. First report of *Giardia duodenalis* in dairy cattle and beef cattle in Shanxi, China. Mol Biol Rep. (2024) 51:403. doi: 10.1007/s11033-024-09342-7, PMID: 38457002

[31] Chen J Zhou L Cao W Xu J Yu K Zhang T. Prevalence and assemblage identified of *Giardia duodenalis* in zoo and farmed Asiatic black bears (*Ursus thibetanus*) from the Heilongjiang and Fujian provinces of China. Parasite. (2024) 31:50. doi: 10.1051/parasite/2024048, PMID: 39212527 PMC11363899

[32] Zhang XX Zhang FK Li FC Hou JL Zheng WB Du SZ. The presence of *Giardia intestinalis* in donkeys, *Equus asinus*, in China. Parasit Vectors. (2017) 10:3. doi: 10.1186/s13071-016-1936-0 28049541 PMC5209919

[33] Wu Y Yao L Chen H Zhang W Jiang Y Yang F. *Giardia duodenalis* in patients with diarrhea and various animals in northeastern China: prevalence and multilocus genetic characterization. Parasit Vectors. (2022) 15:165. doi: 10.1186/s13071-022-05269-9, PMID: 35546681 PMC9097065

[34] Zhao W Li Z Sun Y Li Y Xue X Zhang T. Occurrence and multilocus genotyping of *Giardia duodenalis* in diarrheic and asymptomatic children from south of Zhejiang province in China. Acta Trop. (2024) 258:107341. doi: 10.1016/j.actatropica.2024.107341 39097254

[35] Guo Y Li N Feng Y Xiao L. Zoonotic parasites in farmed exotic animals in China: implications to public health. Int J Parasitol Parasites Wildl. (2021) 14:241–7. doi: 10.1016/j.ijppaw.2021.02.016 33898224 PMC8056123

[36] Abe N Tanoue T Noguchi E Ohta G Sakai H. Molecular characterization of *Giardia duodenalis* isolates from domestic ferrets. Parasitol Res. (2010) 106:733–6. doi: 10.1007/s00436-009-1703-7 20054561

[37] Pantchev N Broglia A Paoletti B Globokar Vrhovec M Bertram A Nöckler K. Occurrence and molecular typing of *Giardia* isolates in pet rabbits, chinchillas, guinea pigs and ferrets collected in Europe during 2006–2012. Vet Rec. (2014) 175:18. doi: 10.1136/vr.102236 24696441

[38] Appelbee AJ Thompson RC Olson ME. *Giardia* and *Cryptosporidium* in mammalian wildlife—current status and future needs. Trends Parasitol. (2005) 21:370–6. doi: 10.1016/j.pt.2005.06.004, PMID: 15982929 PMC7185620

[39] Yun CS Moon BY Lee K Hwang SH Ku BK Hwang MH. Prevalence and genotype analysis of *Cryptosporidium* and *Giardia duodenalis* from shelter dogs in South Korea. Vet Parasitol Reg Stud Rep. (2024) 55:101103. doi: 10.1016/j.vprsr.2024.101103, PMID: 39326959

[40] Kabir MHB Kato K. Examining the molecular epidemiology of *Giardia* and Eimeria species in Japan: a comprehensive review. J Vet Med Sci. (2024) 86:563–74. doi: 10.1292/jvms.23-0525 38556324 PMC11144535

[41] Cao Y Fang C Deng J Yu F Ma D Chuai L. Molecular characterization of *Cryptosporidium* spp. and *Giardia duodenalis* in pet dogs in Xinjiang, China. Parasitol Res. (2022) 121:1429–35. doi: 10.1007/s00436-022-07468-w, PMID: 35233676

[42] Solarczyk P Majewska AC Jędrzejewski S Górecki MT Nowicki S Przysiecki P. First record of *Giardia* assemblage D infection in farmed raccoon dogs (*Nyctereutes procyonoides*). Ann Agric Environ Med. (2016) 23:696–8. doi: 10.5604/12321966.1226869, PMID: 28030946

[43] Ng J Yang R Whiffin V Cox P Ryan U. Identification of zoonotic *Cryptosporidium* and *Giardia* genotypes infecting animals in Sydney’s water catchments. Exp Parasitol. (2011) 128:138–44. doi: 10.1016/j.exppara.2011.02.013 21334325

[44] Minetti C Taweenan W Hogg R Featherstone C Randle N Latham SM. Occurrence and diversity of *Giardia duodenalis* assemblages in livestock in the UK. Transbound Emerg Dis. (2014) 61:e60–7. doi: 10.1111/tbed.12075 23472706 PMC4285228

[45] Xiao HD Su N Zhang ZD Dai LL Luo JL Zhu XQ. Prevalence and genetic characterization of *Giardia duodenalis* and *Blastocystis* spp. in black goats in Shanxi province, North China: from a public health perspective. Animals. (2024) 14:1808. doi: 10.3390/ani14121808 38929427 PMC11201008

[46] Xiao G Qiu Z Qi J Chen JA Liu F Liu W. Occurrence and potential health risk of *Cryptosporidium* and *Giardia* in the three gorges reservoir, China. Water Res. (2013) 47:2431–45. doi: 10.1016/j.watres.2013.02.019 23478072

[47] Cheun HI Kim CH Cho SH Ma DW Goo BL Na MS. The first outbreak of giardiasis with drinking water in Korea. Osong Public Health Res Perspect. (2013) 4:89–92. doi: 10.1016/j.phrp.2013.03.003 24159537 PMC3767097

[48] Alawfi BS. A review on the use of phytochemicals for the control of zoonotic giardiasis. Pak Vet J. (2024) 44:592–8. doi: 10.29261/pakvetj/2024.251

[49] Esch KJ Petersen CA. Transmission and epidemiology of zoonotic protozoal diseases of companion animals. Clin Microbiol Rev. (2013) 26:58–85. doi: 10.1128/CMR.00067-12 23297259 PMC3553666

